# Does Teacher Immediacy Affect Students? A Systematic Review of the Association Between Teacher Verbal and Non-verbal Immediacy and Student Motivation

**DOI:** 10.3389/fpsyg.2021.713978

**Published:** 2021-06-25

**Authors:** Wei Liu

**Affiliations:** College English Department, Xinyang Normal University, Xinyang, China

**Keywords:** verbal immediacy, non-verbal immediacy, students, motivation, systematic review, teachers

## Abstract

In instructional-learning contexts, the relationship between teacher verbal and non-verbal immediacy and student motivation has gained increasing attention. However, no systematic research has been done to review the empirical studies conducted on the impact of teacher immediacy on students' motivation. Hence, the aim of the present study was to systematically review the available literature on different types of teacher immediacy and student motivation. Some common databases were searched and 30 eligible manuscripts were identified. With regard to the key features of the included studies, the review's findings were categorized into different sections, namely “the measures of teacher immediacy employed,” “the measures of student motivation employed,” “designs,” and “educational contexts”. The main findings of the studies were also discussed. The reviewed studies pointed to positive associations between teacher immediacy and student motivation. Finally, limitations of the included studies are discussed and some practical directions for further research are offered, accordingly.

## Introduction

During the past four decades, no construct has gained more attention than teacher immediacy in the field of instructional communication (Madigan and Kim, 2021). Immediacy was first introduced by Mehrabian (1969), who defined this concept as “communication behaviors that enhance closeness to and non-verbal interaction with another” (p. 202). In light of “approach-avoidance theory”, Mehrabian proposed that “people are likely to move toward those they like and away from those they dislike” (Mehrabian, 1971, p. 22). He also distinguished between verbal and non-verbal activities that minimize the perceived physical/psychological intimacy between communicators (Allen et al., 2006). Concerning the significance of immediacy in educational settings, Witt et al. (2004) expounded that verbal and non-verbal behaviors that instructors employ in interactions with their pupils can be deemed as rewarding. These rewarding behaviors can inspire students to become more motivated, attentive, and engaged during a whole session. Richmond et al. (2008) also reported that teachers can minimize students' anxiety, stress, and negative reactions through exhibiting verbal and non-verbal immediate actions.

Besides the aforementioned remarks illustrating the importance of teacher verbal and non-verbal immediacy in general, several scholars (e.g., Yu, 2011; Roberts and Friedman, 2013; Sutiyatno, 2018; Violanti et al., 2018; Sheybani, 2019; Lee, 2020) have pointed to the pivotal role of teachers' immediate behaviors in English as a Foreign Language (EFL)/English as a Second Language (ESL) classrooms. Violanti et al. (2018), for instance, explicated that language teachers' immediate behaviors play a crucial role in the EFL/ESL classrooms because these actions are capable of leading students toward more desirable outcomes. Sheybani (2019) further expounded that teacher immediacy attributes can dramatically enhance EFL/ESL students' willingness to attend classes, which in turn improves their academic achievements. It is mainly due to the fact that “students who attend class regularly have a much greater chance of making high grades” (Moore et al., 2003, p. 325).

Given the importance of teacher immediacy in any educational context (i.e., English language classes and general education courses), numerous studies have sought to examine the association between this interpersonal behavior—immediacy—and student-related factors such as academic engagement, involvement, willingness to attend classes, cognitive learning, affective learning, course retention, satisfaction, and state/trait motivation (e.g., Park et al., 2009; Habash, 2010; Roberts and Friedman, 2013; Faranda, 2015; Gholamrezaee and Ghanizadeh, 2018; Kalat et al., 2018; Pishghadam et al., 2019; Hussain et al., 2020; Derakhshan, 2021). Among different student-related factors, motivation as a prominent factor has received remarkable attention in the field of general education; many studies have investigated the probable correlation between teacher immediacy and student motivation (e.g., Comadena et al., 2007; Velez and Cano, 2008, 2012; Kalish, 2009; Baker, 2010; Littlejohn, 2012; Estepp and Roberts, 2015; Furlich, 2016; Tanriverdi Canbaz and Yavuz, 2016; Barahona Guerrero, 2017; Wijaya, 2017; Stilwell, 2018; Hussain et al., 2021). However, the association between these two variables has remained elusive in English language education (Hsu, 2010; Fallah, 2014).

To deepen our understanding of the depth and breadth of the available literature on teacher immediacy and student motivation, a systematic study is needed to review the empirical studies conducted on this topic. Accordingly, in the present study, the previous research is extended by providing the first systematic review of teacher immediacy and student motivation. It is hoped that this review will contribute to a better understanding of how to enhance students' motivation in instructional-learning contexts, notably EFL,and ESL classrooms.

### Teacher Immediacy

The concept of immediacy, coined by a social psychologist Mehrabian (1969), is defined as “a set of communication behaviors which enhance closeness to and non-verbal interaction with another” (p. 202). Mehrabian worked on non-verbal immediacy at first, but later established a taxonomy of verbal components as well (Averbeck et al., 2006). In terms of his principles of immediacy, Mehrabian submitted “that individuals are attracted to those they like and they ignore or step away from those they dislike” (Allen et al., 2006, p. 24). Immediacy has been attributed to the motivational characteristic of approach-avoidance theory, which states that people approach what they like and avoid what they do not like (Myers et al., 2002; Rocca, 2007).

In 1979, Andersen introduced the application of immediacy to educational environments. She characterized immediacy as a notion that teachers, through the use of certain cues, can reduce the perceived gap between themselves and their students. In this regard, theoretical models posit that teacher immediacy, an interpersonal behavior perceived by students, leads to greater student academic engagement, motivation, and enthusiasm (Hsu, 2010; Marx et al., 2016). Teacher immediacy behaviors (e.g., close proxemics, direct body orientation, smiling, and vocal varieties are all found to be highly effective teaching behaviors (Pogue and AhYun, 2006; York, 2013). Early studies in the field of education named these behaviors as “teacher enthusiasm” or “teacher expressiveness”, while communication scholars referred to them as “immediacy behaviors” (Rocca, 2007). These immediacy behaviors, according to Mehrabian's immediacy taxonomy, can be categorized as verbal and non-verbal behaviors.

#### Verbal Immediacy

Verbal immediacy applies to communication behaviors such as “calling students by names”, “asking for students' feedback about the lessons”, “referring to the class as we and our”, and “engaging in conversations with students before and after class” (Seifu and Gebru, 2012, p. 80). Andersen's (1979) inquiry on the teacher immediacy included behaviors such as discussing outside-of-class experiences, interacting with students before and after classes, inspiring students to actively participate, praising students' work, and offering immediate feedback (Andersen and Andersen, 2005).

#### Non-verbal Immediacy

Non-verbal immediacy is characterized as “communication behaviors that reduce physical and/or psychological distance between teachers and students” (Andersen, 1979, p. 543). These communication behaviors include employing physical gestures, making eye contact, having a relaxed body position, directing body position toward students, and smiling (Chesebro and McCroskey, 2001; Hsu, 2010). Such non-verbal cues enhance the sensory stimulation of interlocutors, resulting in more intense and effective interactions (York, 2013).

### Student Motivation

Pintrich and Schunk (2002) characterized motivation as “the process whereby goal-directed activity is instigated and sustained” (p. 5). Motivation as a communicative mechanism resembles “approach and avoidance motivation”. According to Elliot (1999), approach motivation behavior is prompted by positive consequences, whereas avoidance motivation is instigated by negative results. For instructors, this involves “attempting to give students a reason to be motivated toward a subject by making that subject a desirable event” (Guilloteaux and Dörnyei, 2008, p. 57). As put forward by Katt and Condly (2009), student motivation to learn can be classified into two main categories of “trait motivation” and “state motivation”. Trait motivation is a “general inclination toward learning”, while state motivation is “an attitude toward a particular course” (Trad et al., 2014, p. 138). Although students' trait motivation tends to be relatively constant, their state motivation can be affected by their perceptions of teachers and directly by teachers' actual behaviors (Allen et al., 2006; Katt and Condly, 2009; Dörnyei, 2020; Hiver and Al-Hoorie, 2020). Accordingly, teachers can be active agents within the instructional-learning settings and, therefore, capable of promoting the development of student motivation toward learning.

### Teacher Immediacy and Student Motivation

The most crucial norm in student academic motivation that should be emphasized is the interpersonal behavior of the teacher (Witt and Schrodt, 2006; Ushioda and Dörnyei, 2017; Henry and Thorsen, 2018). Dörnyei and Ushioda (2021) also articulated that teachers are the most important predictors of students' learning motivation. Different theoretical models designed to explain how teacher interpersonal behaviors can influence students propose that teacher immediacy will affect the motivation of students. Of these theories, the Keller's (1987) ARCS model is probably the most relevant theoretical model, describing teacher immediacy as a mediating variable on student motivation. Keller (1987) characterized motivation as requiring four conditions, including “attention”, “relevance”, “confidence”, and “satisfaction”. Among them, getting students' attention is the most crucial factor in motivating students to learn. It is due to the fact that if students do not pay attention, they will not be engaged and will not make effort to learn.

Keller (1987) stated that immediate teachers can enhance their students' motivation because they can address at least three conditions of motivation by employing verbal and non-verbal immediate actions. Initially, immediate teachers gain their students' attention by moving around the class, making eye contact, using vocal variety, and calling students by name. The use of immediate actions can also help teachers to build positive expectancies or confidence in their students. An immediate teacher also seems to generate a positive feeling among students, creating an atmosphere where success appears more likely. Such an atmosphere will in turn make positive expectancies or confidence in students. In regards to satisfaction, Keller (1987) proposed that students who have an immediate teacher are more likely be pleased with their learning experience than those who have a low immediacy teacher.

## Method

### Databases and Search Keywords

Over the period of 7–14 May, 2021, a thorough electronic bibliographic search was performed using some common databases, namely Google Scholar, ERIC, LLBA, ProQuest, Web of Science, PSYCINFO, MEDLINE, and SCOPUS. To locate the related studies, the keywords of “teacher”, “immediacy”, “verbal immediacy”, “non-verbal immediacy”, “student”, and “motivation” were used. The initial search returned 1,030 manuscripts. Following the removal of duplicates and the checking abstracts, 46 manuscripts remained. These manuscripts were evaluated further employing the inclusion and exclusion criteria mentioned hereunder.

### Criteria for Inclusion and Exclusion

Manuscripts were included in the present review if they met the following criteria:

a. Studies measured teacher immediacy (verbal, non-verbal, both);
b. Studies measured student motivation (state, trait, both);
c. Studies were reported or published from inception up to May 2021;
d. Studies were written in English

Manuscripts were excluded if they;

a. Did not assess teacher immediacy
b. Did not assess student motivation

**Figure 1:**
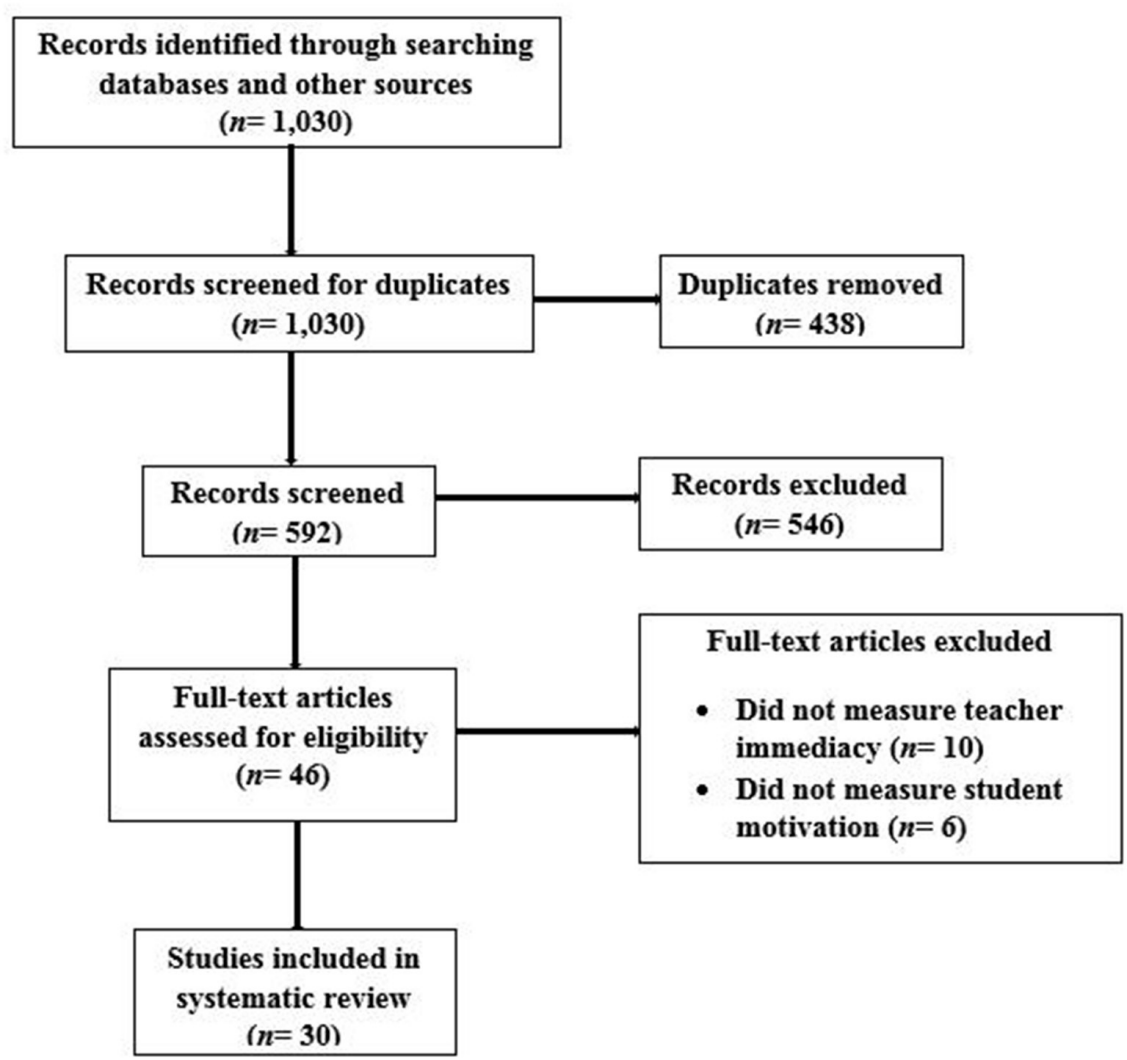
PRISMA diagram demonstrating study selection.

The aforementioned inclusion and exclusion criteria culminated in the inclusion of 30 eligible studies (Figure 1).

### Data Extraction

The included manuscripts were thoroughly reviewed by two researchers. The following information was extracted to summarize the identified studies: (a) publication information, (b) participants demographic information, (c) measure of teacher immediacy, (d) measure of student motivation, (e) Context, (f) main statistical analyses employed, (g) design, and (h) main findings. The derived information is presented in Table 1. Employing Cohen's Kappa, inter-rater reliability was estimated as 0.95 indicated a high degree of consensus between the researchers. The few disagreements were fixed through a consensus between researchers referring to the source data.

**Table 1:** Studies examining teacher immediacy and student motivation.

**References**	**Sample (*N*)**	**Context**	**Immediacy measure**	**Motivation measure**	**Statistical analysis**	**Design**	**Main findings**
Christophel (1990)	Students, teaching assistants, and faculty members	Arts and Sciences classes	“The Immediacy Behavior Scale” (Christophel, 1990)	“Motivation Scale” (Christophel, 1990)	Simple and multiple correlations and regression	Correlational Research	“Positive correlation between verbal and non-verbal immediacy and student motivation/Positive relationship between immediacy and learning”
Frymier (1993)	178 undergraduate students (87 were female, 87 were male, and 4 did not indicate their sex)	Communication courses	“Verbal Immediacy Scale” (Gorham, 1988), “Non-verbal Immediacy Scale” (Richmond et al., 1987)	“Motivation Scale” (Richmond, 1990)	ANOVA with repeated measures, Pearson correlations, Multiple regression, Factorial analysis of variance	Experimental Research (pretest-posttest)	“Student motivation showed positive association with teachers' verbal and non-verbal immediacy”
Christophel and Gorham (1995)	319 students (190 female and 129 male)	Different contexts	“Verbal Immediacy Scale” (Gorham, 1988), “Non-verbal Immediacy Scale” (Richmond et al., 1987)	“Trait/State Motivation Scale” (Christophel, 1990)	Pearson correlation	Test-retest analysis	“A causal relationship between teacher immediacy and student motivation”
Christensen and Menzel (1998)	115 undergraduate students	Not specified!	“Generalized Immediacy Scale” (Andersen, 1979)	“State Motivation Scale” (Christophel, 1990)	One-way ANOVAs	Correlational Research	“Positive relationship between both types of teacher immediacy (verbal and non-verbal) and student motivation/A positive, linear relationships between teacher non-verbal and verbal immediacy and perceived cognitive, affective, and behavioral learning”
Jaasma and Koper (1999)	274 students (172 were female, 100 were male, and 2 did not indicate their sex) Range = 18–60 years (*M* = 23.6)	Not specified!	“Revised Non-verbal Immediacy Measure” (McCroskey et al., 1996), “Verbal Immediacy Scale” (Gorham, 1988)	“Student Motivation Scale” (Rubin et al., 1994)	Pearson product-moment correlation coefficients	Correlational Research	“Positive correlation between verbal and non-verbal immediacy and student motivation”
Cox and Todd (2001)	1,196 students, 12 instructors	Self-contained and Mass-Lecture courses	“Verbal Immediacy Scale” (Gorham, 1988), “Non-verbal Immediacy Scale” (Richmond et al., 1987)	“Student Motivation Scale” (Christophel, 1990)	Pearson product moment correlation coefficients, ANOVA	Correlational Research	“Positive correlation between verbal and non-verbal immediacy and student motivation in both self-contained and mass-lecture formats of the basic communication courses”
Pribyl et al. (2004)	259 students (179 female, 80 male)	Lecture classes	“Non-verbal Immediacy Behaviors Instrument” (Richmond et al., 1987)	“Student Motivation Scale” (Rubin et al., 1994)	Pearson correlation	Correlational Research	“Positive correlation between students' perceptions of teacher non-verbal immediacy, their motivation and cognitive learning”
Jung (2006)	167 students	Business classes	“Non-verbal Immediacy Scale-Observer Report” (Richmond et al., 2003)	“Motivation Scale” (Richmond, 1990)	Pearson's coefficient of correlation	Correlational Research	“Positive and significant association between perceived non-verbal immediacy and perceived course motivation”
Pogue and AhYun (2006)	586 college students (350 were female, 234 were male, and 2 did not report their sex). Mean age= 21.75	General education classes	“Generalized Immediacy Scale” (Andersen, 1979)	“Student State Motivation Scale” (Christophel, 1990)	ANOVA	Experimental Research (factorial design)	“Students experience more motivation with highly immediate teachers/increased achievement is a function of teachers' immediate behaviors”
Comadena et al. (2007)	233 undergraduate students (136 females & 97 males). Range= 18–25 years (*M* = 18.82)	Communication skills course	“Generalized Immediacy Scale” (Andersen, 1979)	“State Motivation Scale” (Christophel, 1990)	ANOVA	Experimental Research (factorial design)	“Teacher immediacy plays an important role in enhancing student motivation and a complimentary role in improving student learning outcomes”
Furlich (2007)	240 undergraduate students	Communication skills course	“Verbal Immediacy Scale” (Gorham, 1988), “Non-verbal Immediacy Scale” (Richmond et al., 1987)	“State Motivation Scale” (Christophel, 1990)	ANCOVA	Correlational Research (descriptive)	“Positive correlation between teacher immediacy and student motivation to learn”
Furlich and Dwyer (2007)	103 undergraduate students	Mathematics classes	“Verbal Immediacy Scale” (Gorham, 1988), “Non-verbal Immediacy Scale” (Richmond et al., 1987)	“State Motivation Scale” (Christophel, 1990)	Multiple linear regression	Correlational Research	“Teachers' verbal and non-verbal immediacy behaviors are positively associated with student motivation”
Zhang and Sapp (2008)	172 college students (114 females & 58 males).	English, business, and communication classes	“Non-verbal Immediacy Measure” (McCroskey et al., 1996)	“State Motivation Scale” (Christophel, 1990)	MANOVA	Experimental Research (factorial design)	“Teachers' non-verbal immediacy behaviors are positively associated with student state motivation and affective learning”
Velez and Cano (2008)	41 undergraduate students	Agriculture course	“Verbal Immediacy Scale” (Gorham, 1988), “Non-verbal Immediacy Scale” (Richmond et al., 1987)	“Approach-Avoidance Motivation Instrument” (Midgley et al., 1998)	Pearson correlation	Correlational Research (descriptive)	“Teacher immediacy has a strong and positive association with students' motivation”
Kalish (2009)	240 undergraduate students (143 were female, 92 were male, and 5 did not report their sex)	General education courses	“Non-verbal Immediacy Scale” (Richmond et al., 1987)	“State Motivation Scale” (Christophel, 1990)	Factorial analysis of variance	Correlational Research	“Teacher non-verbal immediacy has a positive effect on student levels of state motivation”
Baker (2010)	377 undergraduate and graduate students (265 females & 112 males)	Not specified!	“Verbal Immediacy Scale” (Gorham, 1988)	“State Motivation Scale” (Christophel, 1990)	Multiple regression analysis	Correlational Research	“A statistically significant positive relationship between teacher verbal immediacy and student motivation/ A positive association between teacher verbal immediacy and student affective learning”
Hsu (2010)	303 students (259 females & 44 males)	English courses	“Non-verbal Immediacy Scale” (Thomas et al., 1994)	“State Motivation Scale” (Christophel, 1990)	Pearson correlation	Correlational Research	“Teacher non-verbal immediacy has a positive association with students' motivation to learn English”
Littlejohn (2012)	500 high school science students, 32 science teachers	Science courses	“Teacher Communication Behavior Questionnaires” (She and Fisher, 2002)	“State Motivation Scale” (Christophel, 1990)	Pearson correlation, ANOVA	Correlational Research	“Teacher immediacy behaviors lead to increased student motivation”
Kerssen-Griep and Witt (2012)	265 students (144 were female, 121 were male, and 4 did not report their sex) Range= 18–55 years (*M* = 20.04)	Communication courses	“Theoretical taxonomy of TNI” (Richmond et al., 1987)	“State Motivation Scale” (Christophel, 1990)	MANOVA	Experimental Research	“Teachers' non-verbal behaviors play a significant role in increasing student motivation”
Seifu and Gebru (2012)	123 grade 8 students and grade 8 English teachers	English classes	“Verbal Immediacy Scale” (Gorham, 1988), “Non-verbal Immediacy Scale” (Thomas et al., 1994)	“Student Motivational State Questionnaire” (Guilloteaux, 2007)	Pearson correlation	Correlational Research (descriptive)	“Positive correlation between teacher immediacy and student motivation to learn English”
Velez and Cano (2012)	208 students (36% were female and 64% were male)	Agricultural and Environmental sciences courses	“Verbal Immediacy Scale” (Gorham, 1988), “Non-verbal Immediacy Scale” (Richmond et al., 1987)	“Motivated Strategies for Learning Questionnaire” (Pintrich et al., 1991)	Pearson product–moment correlations	Correlational Research (descriptive)	“Verbal immediacy has a high correlation with student motivation to do the tasks”
Fallah (2014)	252 Iranian EFL learners (151 were female and 101 were male) Range= 18-43 years (*M* = 20.71)	English classes	“Verbal Immediacy Scale” (Gorham, 1988), “Non-verbal Immediacy Scale” (Richmond et al., 1987)	“Motivation Scale” (Gardner et al., 1985)	Structural equation model (SEM)	Correlational Research	“Teacher immediacy has a significant positive effect on the learners' motivation”
Estepp and Roberts (2015)	306 undergraduate students (63.7% were female and 36.3% were male)	Agricultural and Life sciences courses	“Immediacy Behavior Scale” (Christophel, 1990)	“Motivated Strategies for Learning Questionnaire” (Pintrich et al., 1991)	Pearson correlation	Correlational Research (descriptive)	“Positive correlation between teacher immediacy and student motivation”
Furlich (2016)	77 undergraduate students	Communication courses	“Verbal Immediacy Scale” (Gorham, 1988), “Non-verbal Immediacy Scale” (Richmond et al., 1987)	“State Motivation Scale” (Christophel, 1990)	Independent linear regression	Correlational Research	“A significant linear regression relationship between teacher verbal immediacy and student motivation”
Tanriverdi Canbaz and Yavuz (2016)	221 students Range= 17-25 years	Schools	“Teacher Immediacy Behaviors Questionnaire” (Tanriverdi Canbaz and Yavuz, 2016)	“Student Motivation Questionnaire” (Tanriverdi Canbaz and Yavuz, 2016)	ANOVA	Correlational Research	“A considerable difference between the motivation scores of the students with the lower immediacy perception and those of the students with the higher immediacy perception scores”
Barahona Guerrero (2017)	139 undergraduate students (23.7% female, 76.3% male) Range= 18–39 years, *M* = 20.9	Engineering classes	“Verbal Immediacy Scale” (Gorham, 1988), “Non-verbal Immediacy Scale” (Richmond et al., 1987)	“State Motivation Scale” (Christophel, 1990)	A multiple linear regression	Correlational Research	“Teacher verbal and non-verbal immediacy predict student motivation”
Wijaya (2017)	60 students	X IIS 2/XI MIA 4	Questionnaire, Group Interview, Observation	Questionnaire, Group Interview, Observation	Simple descriptive statistic, Interactive model of data analysis	Mixed Method Research	“Teacher non-verbal immediacy enhances students' motivation to learn English”
Stilwell (2018)	530 students	Schools	“Verbal Immediacy Scale” (Gorham, 1988), “Non-verbal Immediacy Scale Observer Reports” (Richmond et al., 2003)	“Teacher Rating of Academic Achievement Motivation” (Stinnett et al., 1991)	Pearson correlation	Correlational Research	“Positive relationship among teacher immediacy, student motivation, and cognitive learning”
Megawati and Hartono (2020)	EFL teachers and students	English classes	Interview, Observation	Interview, Observation	Simple descriptive statistic, Interactive model of data analysis	Qualitative research	“Students' motivation are affected by both teachers' verbal and non-verbal communication behaviors, notably questions, and facial expressions”
Hussain et al. (2021)	726 students	General education courses	“Verbal Immediacy Scale” (Gorham, 1988), “Revised Non-verbal Immediacy Measures” (McCroskey et al., 1996)	“Students Motivation Scale” (Rubin et al., 1994)	Pearson correlation	Correlational Research (descriptive)	“A strong correlation between verbal and non-verbal teacher immediacy and student motivation”

## Results

The review's findings were listed according to the key features of the included manuscripts, including “the measures of teacher immediacy employed”, “the measures of student motivation employed”, “designs”, and “educational contexts”. Afterwards, the main findings of the manuscripts were discussed based on different types of teacher immediacy: (1) Verbal immediacy, (2) Non-verbal immediacy, and (3) Both verbal and non-verbal immediacy.

### Measures of Teacher Immediacy

As shown in Table 1, most of the included studies (60%) utilized “Verbal Immediacy Scale” (Gorham, 1988) and/or “Non-verbal Immediacy Scale” (Richmond et al., 1987) to measure teacher immediacy. The rest (40%) assessed teacher immediacy through observation, group interview, and other reliable scales such as “Generalized Immediacy Scale” (Andersen, 1979), “Immediacy Behavior Scale” (Christophel, 1990), “Revised Non-verbal Immediacy Measure” (McCroskey et al., 1996), and “Non-verbal Immediacy Scale-Observer Report” (Richmond et al., 2003).

### Measures of Student Motivation

Table 1 delineates that the majority of studies (53%) used “Motivation Scale” (Christophel, 1990) to measure student motivation. The remaining studies (47%) evaluated student motivation via observation, interview, and other scales such as “Student Motivation Scale” (Rubin et al., 1994), “Motivation Scale” (Richmond, 1990), “Motivated Strategies for Learning Questionnaire” (Pintrich et al., 1991), “Motivation Scale” (Gardner et al., 1985), and “Student Motivational State Questionnaire” (Guilloteaux, 2007).

### Educational Contexts (General Education and English Language Education)

**Figure 2:**
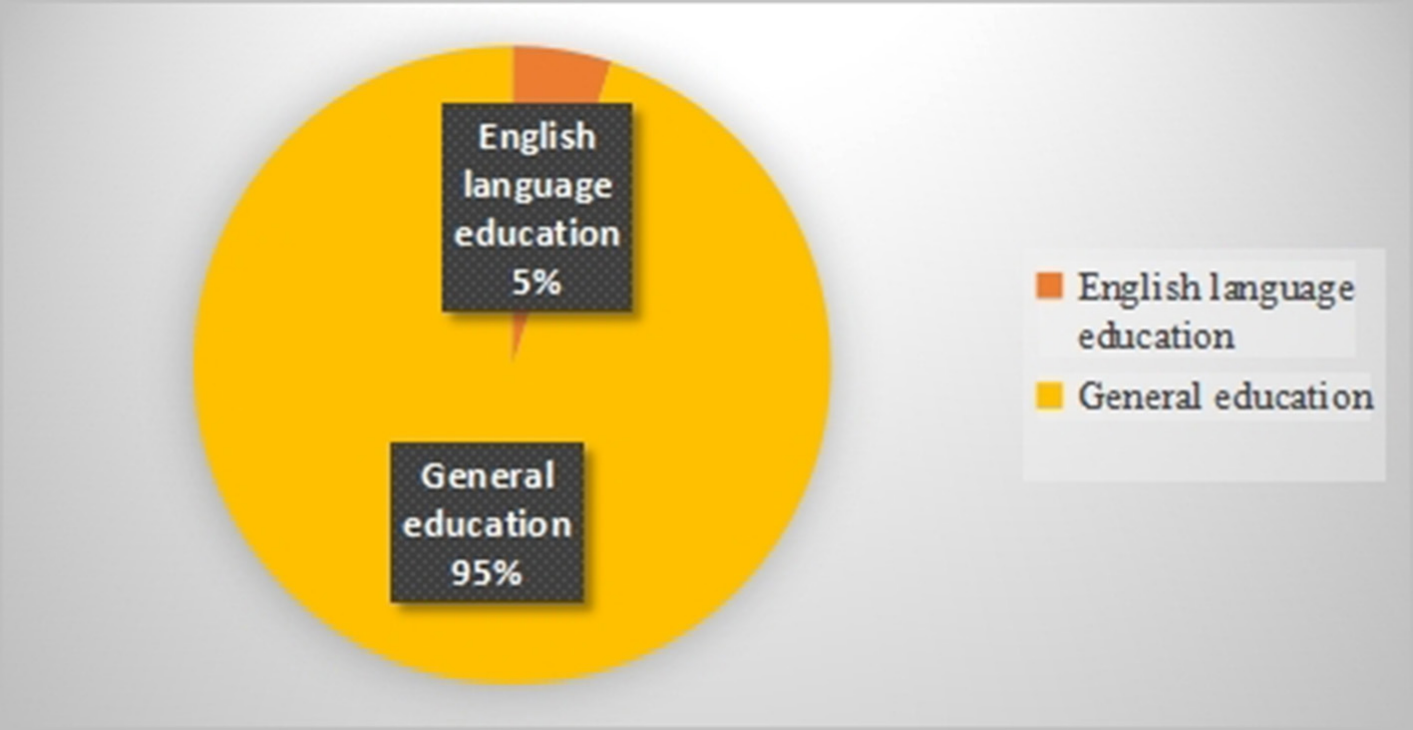
Educational contexts.

Of 30 included studies, only 5 empirical studies (17%) were carried out in EFL/ESL classes, the rest (83%) examined the interplay of teacher immediacy and students' motivation in general educational contexts such as science classes, engineering classes, communication courses, business courses, agricultural courses. That is, the consequences of English teachers' immediate behaviors for EFL/ESL students' academic motivation received scant attention (Figure 2).

### Study Designs

**Figure 3:**
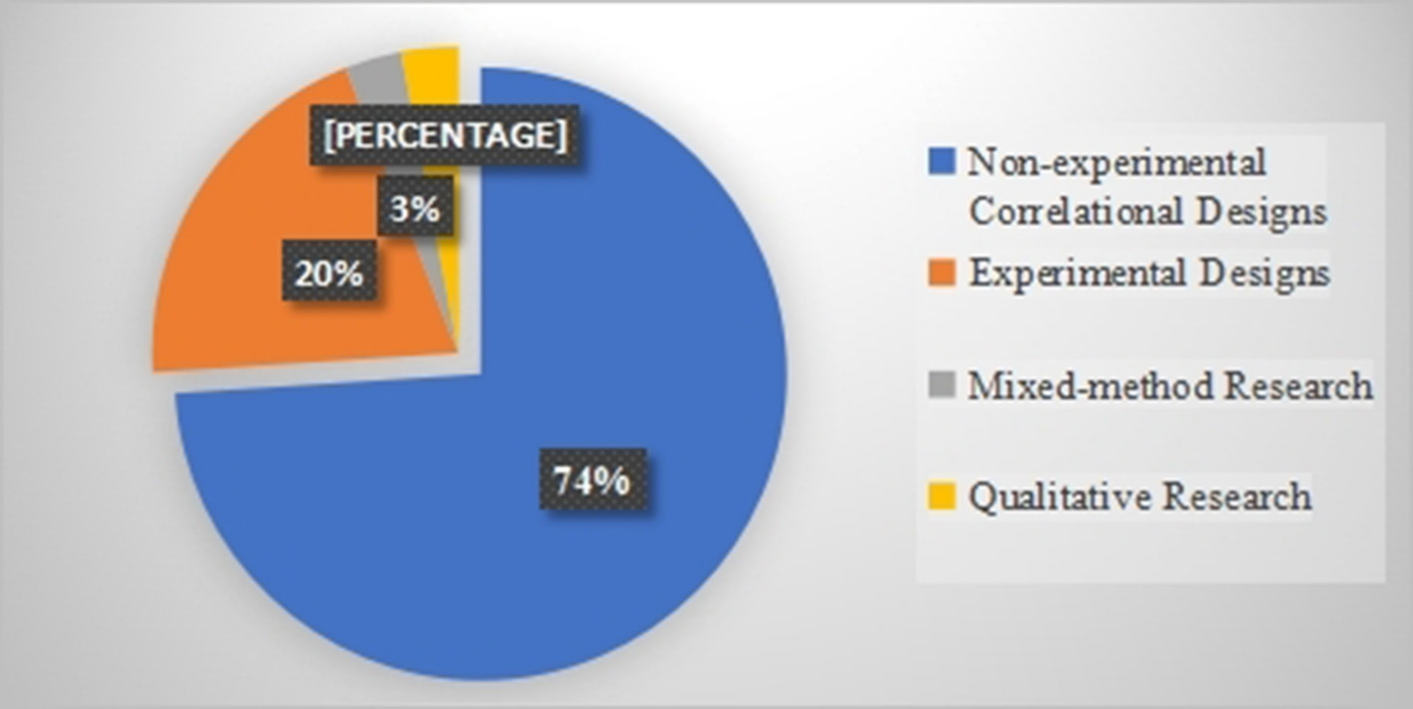
Designs of the included studies.

As shown in Figure 3, several studies (74%) in the current systematic review utilized correlational designs (non-experimental). The rest used experimental (20%), qualitative (3%), and mixed-methods research (3%) in studying teacher immediacy and student motivation.

### Teacher Immediacy and Student Motivation

#### Teacher Verbal Immediacy and Student Motivation

Among the included studies, only one study (Baker, 2010) investigated the relationship between teacher verbal immediacy and student motivation. In doing so, 699 graduate and undergraduate students voluntarily completed two questionnaires, namely “Verbal Immediacy Scale” (Gorham, 1988) and “State Motivation Scale” (Christophel, 1990). Analyzing students' perceptions, Baker (2010) found that students perceive teachers' immediacy behaviors as an important motivational factor in instructional-learning environments.

#### Teacher Non-verbal Immediacy and Student Motivation

Of 30 studies included in this review, seven studies (Pribyl et al., 2004; Jung, 2006; Zhang and Sapp, 2008; Kalish, 2009; Hsu, 2010; Kerssen-Griep and Witt, 2012; Wijaya, 2017) examined the role of teacher non-verbal immediacy in students' motivation. The findings indicated that teachers' non-verbal behaviors play a significant role in enhancing student motivation. To put differently, the results of these studies revealed that teacher non-verbal immediacy is a strong predictor of student motivation.

#### Teacher Verbal and Non-verbal Immediacy and Student Motivation

The majority of reviewed studies (*n* = 22) probed the association between teacher verbal and non-verbal immediacy and student motivation to learn. Of these studies, 20 studies found a positive relationship between both verbal and non-verbal immediacy and student motivation. In other words, they reported that students' level of motivation can be remarkably enhanced by their teachers' verbal and non-verbal immediacy. On the other hand, two studies (Velez and Cano, 2012; Furlich, 2016) found that only teacher verbal immediacy can lead to increased student motivation.

## Discussion

The current study sought to offer the first systematic review on teacher verbal and non-verbal immediacy and student motivation. By reviewing, summarizing, and analyzing the relevant studies on this topic, it is hoped to shed more light on the significance of teacher immediacy for students, on the one hand, and to develop a broader picture of the current state of the art, on the other hand. This section discusses the main findings and crucial points. In light of these key findings, the limitations of the included studies are highlighted, and some practical directions for future research are delineated.

### Main Findings

While research has substantiated that teacher immediacy has numerous positive effects on teachers (e.g., Teven and Hanson, 2004; Santilli et al., 2011; Kelly and Westerman, 2014; Lybarger et al., 2017; Kalat et al., 2018; Frymier et al., 2019; Nayernia et al., 2020), the results indicate that the effects may also apply to their students. One of the most outstanding results is that there was some indication that teacher immediacy is tied with increased student motivation. Students being instructed by a teacher using verbal and non-verbal immediacy behaviors are more motivated than those instructed by teachers not employing immediacy actions. This finding may be explained by the fact that getting students' attention is the most crucial factor in motivating students. Moving around the class, making eye contact, and calling students by name enable teachers to do so (Keller, 1987). Another possible explanation for this is that those teachers tend to enhance their students' state motivation may strengthen their interaction with them (Allen et al., 2006; Myers et al., 2014).

Furthermore, we noticed some indications that immediacy behaviors will influence students beyond their motivation and probably interact with their learning outcomes—with moderate correlations identified with higher students' achievements. A probable explanation for this might be that the immediacy behaviors that teachers exhibit in interactions with students can inspire students to become more attentive, which in turn improves students' achievements (Mazer, 2013; York, 2013; Ai and Giang, 2018).

### Limitations of the Included Studies

A number of limitations need to be noted regarding the included studies. First, it was found that all studies merely employed observer-report questionnaires to measure teacher immediacy; hence, the voices/perceptions of teachers regarding their immediacy are not heard. Second, all but one study (Wijaya, 2017) relied solely on questionnaires to measure teacher immediacy and student motivation. Third, most studies (74%) utilized non-experimental correlational designs; the experimental designs received scant attention. Forth, a scant number of studies have examined the role of teacher immediacy in school students' motivation. To put it differently, most of the included studies were conducted in universities. Fifth, the majority of studies (95%) focused on the impact of teacher immediacy on general education courses. That is, a limited number of studies probed the consequences of teacher immediacy in EFL/ESL classes. Finally, the studies included in this review scarcely examined the mediating effects of situational variables (e.g., age, gender, academic degree, etc.) on the relationship between teacher immediacy and student motivation.

### Directions for Future Research

In light of the critical evaluation of the studies presented in this review, some suggestions for future research are provided to deepen the current understanding of the topic. Given the importance of the topic, first and foremost, more empirical studies examining the associations between teacher immediacy and student motivation are needed. While the included studies provide some indications for the aforementioned associations, more extensive research in terms of design, samples, and findings is required. Moreover, given the scarcity of studies investigating the consequences of language teacher immediacy for EFL/ESL students' motivation, future research needs to be carried out to establish whether present findings will be generalized to English language classes.

A further practical direction for future work pertains to the measurement of teacher immediacy. As the voices of teachers regarding their immediacy are not heard, it would be interesting to provide different perspectives on this phenomenon beyond observer-report scales. Additionally, more research is needed to consider the role of situational variables. Future studies are highly recommended to determine whether different situational variables (e.g., age, gender, academic degree, etc.) might moderate the effects of teacher verbal and non-verbal immediacy on student motivation. Personality traits should also be taken into consideration in future research.

### Implications for Practice

The findings of the present review can be informative and beneficial for teachers in all instructional-learning contexts (i.e., English language classes and general education courses). Employing verbal and non-verbal actions, teachers can increase the psychological intimacy between themselves and their students, which contributes to increased student motivation (Averbeck et al., 2006; Richmond et al., 2008). A higher degree of students' motivation can increase their learning outcomes, which is the ultimate goal of any educational system (Allen et al., 2006; Ai and Giang, 2018; Gholamrezaee and Ghanizadeh, 2018; Pishghadam et al., 2019; Derakhshan, 2021). Furthermore, the review's outputs may also be informative for teacher educators. They should highlight the significance of teachers' interpersonal variables, especially verbal non-verbal immediacy to assist both pre- and in-service teachers in enhancing the amount of students' trait and state motivation. Being aware of the significance of verbal and non-verbal immediacy behaviors, teachers can also provide more efficient and appealing instruction. Hence, in teacher training courses, both pre- and in-service teachers should be equipped with the knowledge of appropriate immediacy behaviors (e.g., close proxemics, smiling, vocal varieties, etc.) to take advantage of these actions in their classrooms.

## Conclusions

To sum up, the current systematic review has shed more light on the association between teacher verbal and non-verbal immediacy and student motivation, raising several concerns that have not been addressed in this area. To the best of our knowledge, this is the first systematic review focusing on the relationship between teacher immediacy and student motivation in instructional-learning contexts. The findings indicate that immediate teachers are capable of enhancing students' motivation. Based on the key features of the included studies, it is concluded that further research adopting more robust designs, employing self-report questionnaires, and examining the mediating effects of situational variables are required. Moreover, with regard to the number of studies conducted on the influence of immediate behaviors on EFL and ESL students' academic motivation, it is reasonable to infer that this area is still in its infancy and needs much more attention.

## Data Availability Statement

The original contributions presented in the study are included in the article/supplementary files, further inquiries can be directed to the corresponding authors.

## Author Contributions

The author confirms being the sole contributor of this work and has approved it for publication.

## Conflict of Interest

The author declares that the research was conducted in the absence of any commercial or financial relationships that could be construed as a potential conflict of interest.
